# Cohort Profile: The Oxford Biobank

**DOI:** 10.1093/ije/dyx132

**Published:** 2017-07-31

**Authors:** Fredrik Karpe, Senthil K Vasan, Sandy M Humphreys, John Miller, Jane Cheeseman, A Louise Dennis, Matt J Neville

**Affiliations:** 1 Oxford Centre for Diabetes, Endocrinology and Metabolism, University of Oxford; 2 Oxford National Institute for Health Research, Biomedical Research Centre, Churchill Hospital, Oxford, UK

**OBB in a nutshell**

- The Oxford Biobank is a population-based repository of biological material and health-related information on ∼8000 healthy participants, men and women aged 30–50 years, from Oxfordshire, UK.
- The bioresource includes a broad range of cardiovascular- and obesity-related phenotypes including biochemical and genetic biomarkers, anthropometric measurements and body composition assessed using dual energy X-ray absorptiometry.
- The cohort has the specific feature to allow for future dedicated recall studies based on baseline phenotype and genotype.
- With that capacity, the Oxford biobank is a resource for mechanistic research of genetic and phenotypic traits in a broad range of chronic disease such as cardiovascular disease, type 2 diabetes and obesity complications.
- Researchers interested in using the cohort should go through the online portal [www.oxfordbiobank.org.uk].

## Why was the cohort set up?

Major progress has been made over the past decade in the understanding of the genetic background to chronic metabolic disease such as type 2 diabetes (T2D) and atherosclerotic cardiovascular disease (CVD). These disorders show a significant degree of heritability and disease pathogenesis that rely on the combination of a multitude of unfavourable genotypes on which over-nutrition, lack of physical exercise, obesity and smoking augment the phenotype. Currently, the number of common genetic variants robustly associated with CVD and T2D are increasing with the increasing size of discovery cohorts; for CVD, the number now exceeds 50 variants^1–3^ and for T2D and glycaemic traits, the corresponding number is about 75.^4^^,^^5^ Combining several genome-wide association studies (GWAS) datasets which include information on highly relevant intermediate phenotypes has potentially helped in discovery and replication of several disease loci and identification of novel pathways and pleiotropic genes. However, little is known about the functional consequences of most of the identified gene variants. The use of well-characterized bioresources, in which investigations into intermediate phenotypes can be performed, will be invaluable in order to provide mechanistic insight into these poorly characterized genes and thus promote translational research.

To this end the Oxford Biobank (OBB) was set up with the primary goal of establishing a local cohort accessible for genomic translational research. The resource is built to enable studies on physiological consequences of genetic mechanisms of disease. A leading principle has been to seek informed consent from participants to be re-approached for future discrete projects. Therefore, based on the information gathered during a baseline visit, ‘recruit-by-genotype’ (RbG) and ‘recruit-by-phenotype’ (RbP) projects allow for detailed investigations of associations between genotypes and biomarkers, or monitoring of more detailed physiological processes. The OBB serves as a resource for researchers to investigate mechanisms leading to increased T2D and CVD susceptibility and to explore novel therapeutic targets in the prevention and treatment of chronic non-communicable diseases.

## Who is in the cohort?

The OBB is a random, population-based recruitment of healthy participants between the ages of 30 and 50 years from the Oxfordshire general population (approximately 800 000 inhabitants). Individuals with: previous diagnosis of myocardial infarction or heart failure currently on treatment; untreated malignancies; or other systemic ongoing disease, and pregnant women were excluded from participation. The OBB recruitment began in 1999 and includes 7640 (4316 women and 3324 men) individuals as of October 2016, with the aim of having a local cohort of 10 000 people among whom recalling can be achieved. This sample size is based on the ability to identify an average of 25 people who are homozygous for what is normally considered common genetic variants (minor allele frequency greater than 0.05). For the purpose of reaching out to even larger populations to allow for recruitment of carriers of rare gene variants or phenotypes, the Oxford Biobank is a partner of the National Institute of Health Research (NIHR) Bioresource currently reaching ∼100 000 people. Baseline demographics of the OBB participants are provided in Table 1.

**Table 1:** Baseline characteristics of the OBB participants

Characteristics	*n*	Male (*n* = 3324)	*n*	Female (*n* = 4316)
Sociodemographics				
Age	3324	43 (37, 46)	4316	42 (37, 46)
Smoking^a^				
Never+Ex-smoker	3315	2901 (87.5)	4308	3929 (91.2)
Current smoker		414 (12.5)		379 (8.8)
Alcohol intake^a^				
No alcohol	3315	26 (0.8)	4308	138 (3.2)
Moderate		2865 (86.4)		3803 (88.3)
Heavy		424 (12.8)		367 (8.5)
Physical activity^a^				
Sedentary	3315	149 (4.5)	4308	178 (4.1)
Moderate		1983 (59.8)		3154 (73.2)
Vigorous		1183 (35.7)		976 (22.7)
Menopause^a^		–	3412	266 (7.8)
Anthropometry				
Height (cm)	3322	179 (174, 183)	4315	165 (161, 170)
Weight (kg)	3322	83.5 (75.5, 93.0)	4315	66.5 (59.8, 75.7)
Body mass index (kg/m^2^)	3322	26.1 (23.8, 28.7)	4315	24.1 (21.9, 27.6)
Waist circumference (cm)	3317	92 (85, 99)	4307	80 (73, 88)
Hip circumference (cm)	3292	101 (97, 106)	4306	100 (95, 106)
Supra-iliac skinfold thickness (mm)	3320	17 (12, 25)	4308	17 (11, 26)
Subscapular skinfold thickness (mm)	3283	17 (13, 22)	4271	18 (12, 24)
Triceps skinfold thickness (mm)	3312	12 (9, 18)	4309	22 (17, 28)
Biceps skinfold thickness (mm)	3324	7 (5, 10)	4316	12 (8, 18)
Thigh skinfold thickness (mm)	1388	14 (10, 20)	2263	35 (24, 53)
Systolic blood pressure (mmHg)	3324	126 (119, 134)	4316	114 (107, 123)
Diastolic blood pressure (mmHg)	3324	79 (73, 85)	4316	73 (67, 79)
Biochemical tests				
Fasting glucose (mmol/l)	3317	5.3 (5.1, 5.7)	4307	5.0 (4.8, 5.3)
Fasting insulin (mU/l)	3293	12.5 (9.6, 16.3)	4271	11.0 (8.5, 14.3)
Total cholesterol (mmol/l)	3317	5.3 (4.6, 6.0)	4305	5.0 (4.4, 5.7)
Triglycerides (mmol/l)	3317	1.2 (0.8, 1.7)	4305	0.8 (0.6, 1.1)
HDL-cholesterol (mmol/l)	3317	1.2 (1.0, 1.4)	4305	1.5 (1.2, 1.8)
LDL-cholesterol (mmol/l)	3286	3.4 (2.9, 4.1)	4301	3.1 (2.6, 3.6)
Apolipoprotein B (g/l)	3317	1.0 (0.8, 1.1)	4305	0.8 (0.7, 1.0)
Apolipoprotein A1 (g/l)	2103	1.3 (1.2, 1.5)	2509	1.5 (1.3, 1.7)
NEFA (µmol/l)	3315	404 (286, 54)	4303	489 (346, 658)
hs-CRP (mg/l)	3305	0.6 (0.2, 1.7)	4290	0.5 (0.1, 1.8)
3-hydroxy butyrate (umol/l)	3314	51.2 (34.9, 85.7)	4307	64.1 (38.9, 116.2)
Lactate (mmol/l)	3290	0.85 (0.66, 1.13)	4284	0.67 (0.54, 0.94)
Glycerol (µmol/l)	3310	37.9 (27.5, 52.6)	4294	56.5 (40.3, 77.9)
IGF-1 (µg/l)	1148	208 (177, 246)	1154	205 (167, 247)
IGFBP-1 (µg/l)	1147	30 (19, 45)	1154	44 (28, 63)
DXA measurements				
Fat mass (kg)				
Arms	2146	2.2 (1.7, 2.8)	2871	2.6 (2.0, 3.3)
Legs	2146	6.0 (4.8, 7.5)	2871	8.5 (6.8, 10.7)
Trunk	2146	12.7 (9.1, 16.8)	2871	10.7 (7.6, 14.9)
Android	2146	2.1 (1.4, 2.9)	2871	1.6 (1.0, 2.4)
Gynoid	2146	3.3 (2.6, 4.1)	2871	4.3 (3.4, 5.4)
Total fat	2146	22.0 (16.9, 28.1)	2871	22.6 (17.6, 29.6)
Visceral fat	2146	0.9 (0.5, 1.6)	2871	0.3 (0.1, 0.6)
Lean mass(kg)				
Arms	2146	7.3 (6.6, 8.2)	2871	4.2 (3.8, 4.7)
Legs	2146	19.9 (18.2, 21.8)	2871	13.9 (12.6, 15.3)
Trunk	2146	26.9 (24.9, 29.0)	2871	20 (18.4, 21.6)
Android	2146	4.0 (3.6, 4.3)	2871	2.9 (2.6, 3.2)
Gynoid	2146	9.1 (8.4, 10.1)	2871	6.4 (5.9, 7.0)
BMD (g/cm^2^)*				
Total	2146	1.1 (1.0, 1.2)	2871	1.2 (1.0, 1.3)
Spine	2146	1.2 (1.1, 1.3)	2868	1.1 (1.0, 1.2)

IGF-1, insulin-like growth factor-1; IGFBP-1, insulin-like growth factor binding protein-1; hs-CRP, highly sensitive C-reactive protein; BMD, bone mineral density.

All data presented as median (interquartile range) and ^a^frequency (percentage).

^a^Smoking: classified as ex-smokers and current smokers.

^a^Alcohol intake: moderate consumption, less than 21 units in men and less than 14 units in women (per week); heavy consumption, greater than 21 units in men and greater than 14 units in women (per week).

^a^Physical activity classified as moderate and vigorous activity per week.

### Recruitment

The OBB includes a randomized, age-stratified sample obtained from Oxfordshire and the Thames Valley. The Thames Valley Primary Care Agency has enabled random recruitment by providing lists of Oxfordshire residents registered with a local general practitioner and aged 30–50 years. An invitation letter along with the study information and response sheet were sent to all the participants. Subjects who expressed willingness to enrol in the OBB were contacted by telephone or e-mail, in order to convey a brief overview of the study aims and objectives, by trained research nurses. Possible exclusions for active disease or previous history of T2D or CVD were confirmed during this contact, and only eligible participants were scheduled for a clinic visit. Eligible participants were then scheduled to visit the Clinical Research Unit at the Oxford Centre for Diabetes, Endocrinology and Metabolism for a baseline investigation. Exclusion criteria were type 1 and type 2 diabetes, established CVD, cancer, known autoimmune or severe inflammatory conditions, substance abuse or psychiatric condition making participation in Stage 2 (see later) unlikely. The OBB protocol is approved by the Oxfordshire Clinical Research Ethics Committee (08/H0606/107+5) and all participants have provided informed consent.

## How often have they been followed up?

All participants have a detailed baseline characterization (Stage 1). Subsequently, selected volunteers are invited for a second visit (recall) to comply with a specific research protocol (Stage 2). Information on who is selected for such recall studies will be determined by the research question and the available information from the Stage 1 visit. Such recalls could be either ‘recall-by-genotype’ or ‘recall-by-phenotype’.

## What has been measured? 

The OBB has collected a broad range of metabolic-, CVD- and obesity-related phenotypes based on blood plasma phenotyping, genetic biomarkers, questionnaires, anthropometric measurements and body composition assessment using dual-energy X-ray absorptiometry (DXA). A brief description of variables collected at baseline is provided below.

### 

#### 

##### Anthropometry

This included height, weight, waist and hip circumference (WC and HC) measurements, and calliper-measured skinfold thickness of the upper arm (over biceps and triceps), subscapular, abdominal and thigh regions.

##### Questionnaire-based assessments

Information on potential risk exposures or confounders in disease pathology, such as physical activity, smoking and alcohol intake, were obtained using validated questionnaires. The OBB participants were also interviewed by trained nurses on family history of any chronic disease (such as the ‘Rose’ questionnaire for angina pectoris) given that the family history is a well-known predictor of CVD and T2D. The questionnaires were all adopted from previously used studies and have not been internally validated.

##### Blood pressure

An automatic pulse-detecting sphygmomanometer (Omron M3) was used to record systolic and diastolic blood pressure, using a standard protocol involving four sequential measurements after 10 min in the semi-recumbent position. The average of the last three measurements was used.

##### Biochemistry

Venous antecubital blood was drawn after an overnight fast and immediately put on ice. Plasma was separated within 60 min, frozen at −20°C within 120 min and transferred to −80°C within 4 h. Plasma samples have been analysed for glucose, lipids/lipoproteins (cholesterol, triglycerides, high-density lipoprotein (HDL) cholesterol, apolipoprotein-B (ApoB), apolipoprotein A1, C-reactive protein (CRP), insulin, total non-esterified fatty acids (NEFA), glycerol, 3-hydroxybutyrate and lactate. A subset of samples have been analysed for insulin-like growth factor (IGF-1) and insulin-like growth factor binding protein-1 (IGFBP-1) (*n* = ∼2200). Details of the platforms used for biochemical analysis are provided in Table 2. Adiponectin is currently being analysed in all participants. A biorepository of aliquots (10–15 x 0.5 ml of both EDTA- and heparin-anticoagulated plasma as well as serum) is stored for future use.

**Table 2:** List of platforms used for biochemical tests

Biochemical tests	Analysis method/platform used
Fasting glucose	Analysed using Instrumentation Laboratory IL Test^TM^ kits on an ILab 600/650 clinical chemistry analysers (Werfen, Warrington, UK)
Total cholesterol	
Triglycerides	
HDL- and LDL-cholesterol	Analysed using Randox kits adapted for use on the Ilab 600/650 analysers (Randox Laboratories, Crumlin, Northern Ireland)
Non-esterified fatty acids (NEFA)	
Apolipoprotein-B	
Apolipoprotein A1	
Glycerol	
Lactate	
3-hydroxybuthyrate	
C-reactive protein	Analysed using a Siemens ADVIA wide range CRP kit adapted for use on the Ilab 600/650 analysers. (Siemens Healthcare Diagnostics, Camberley, UK)
Fasting insulin	Millipore Human Insulin specific radioimmunoassay (Millipore UK, Watford, UK)
Adiponectin	Perkin Elmer AlphaLisa Human Adiponectin kit (Waltham, MA, USA)

##### Metabolomics

The NMR-based metabolomics platform data containing ∼230 metabolites^6^ has been performed on ∼7100 Oxford biobank plasma samples. Additionally, the mass spectroscopy-based technology Metabolon^®^ is available on a select set of 2250 samples on whom detailed DXA-acquired body composition data are available to study the association between specific fat depots and metabolome

##### Genomics

For each OBB participant, 3 × 5-ml aliquots of whole blood are collected and frozen at −80°C for isolation of genomic DNA. Single nucleotide polymorphism (SNP) array data have been generated using the Illumina Infinium Human Exome Beadchip 12v1 array platform for the first consecutive 5900 DNAs, and Affymetrix UK Biobank Axiom Array chip on the first consecutive 7500 participants. Beyond this, high throughput custom genotyping is facilitated by DNA being plated into 384-well format for typing on an Applied Biosystems 7900HT analyser using Applied Biosystems Taqman^®^ SNP genotyping chemistries, or by LGC Genomics KASP™ custom assays using KASP genotyping chemistry.

##### Body composition and bone mineral density assessment

Body composition is assessed using GE Lunar iDXA and all data are analysed with Encore software (version 11.0; GE. Medical Systems, Madison, WI, USA), which beyond regional body composition also includes an algorithm for quantification of visceral adipose tissue (VAT).

## What has it found? Key findings and publications

The specific feature of the OBB is that all participants have provided informed consent to be re-contacted for follow-up studies. The cohort has therefore been used for both cross-sectional analyses as well as dedicated follow-up studies.

### 

#### 

##### Findings from cross-sectional studies from the baseline data

The 7640 participants recruited so far in the OBB have a wide range of phenotypes that allow studying specific disease characteristics in relation to both their genotype and their phenotype. The percentages of various incident phenotypes at baseline, such as impaired fasting glucose (IFG), insulin resistance (IR), undiagnosed T2D and hypertension, overweight and obesity, are provided in Table 3. Results from various study designs are summarized below.

**Table 3:** Prevalence of incident cardio-metabolic phenotypes at baseline screening

Phenotype	Total n*	Male *N* (%)	Total n*	Female *N* (%)
Impaired fasting glucose	3317	983 (29.6)	4307	439 (10.2)
Hypertension	3324	610 (18.4)	4316	298 (6.9)
Hypertriglyceridaemia	3317	821 (24.6)	4307	295 (6.9)
Overweight	3277	1486 (45.4)	4268	1171 (27.4)
Obesity	3277	572 (17.4)	4268	641 (15.0)

Impaired fasting glucose: defined as fasting glucose ≥ 5.6 mmol/l. Hypertension: defined as systolic blood pressure ≥ 141 or diastolic blood pressure ≥ 90 mmHg. Hypertriglyceridemia: defined based on ATP III cut-off of >1.7 mmol/l. Overweight: defined as BMI ≥ 25.0 to <29.9 kg/m^2^. Obesity: defined as BMI ≥ 30.0 kg/m^2^.

*N based on number of individuals for whom baseline values are available.

##### Genome-wide association studies (GWAS)

The focus of some of the key findings in the GWAS included identification of novel genetic variants associated with various disease-related phenotypes such as obesity, T2D, hyperglycaemic and hyperinsulinaemic traits, anthropometric traits, fat distribution and blood pressure.^3^^,^^7–19^ This was facilitated by collaborating with several international GWAS consortia such as the WTCCC, DIAGRAM, GIANT and the MAGIC consortia. Such efforts have helped identify several novel genetic variants associated with T2D,^18^ adiposity^7^^,^^13^^,^^16^^,^^20^^,^^21^ and CVD traits.^14^^,^^22^ Notably, the discovery of rs9939609 variant located in the first intron of *FTO* (fat mass- and obesity-associated) gene that predisposes to diabetes through an effect on body mass index (BMI),^23^ and the *MC4R* (melanocortin-4 receptor) genetic variant in common obesity risk,^24^ were early contributions of OBB data to obesity genetics.

##### Cross-sectional observational studies

The paradoxical association between upper body android and lower body gluteofemoral fat with CVD and T2D traits was shown using precise estimates of fat depot measured by DXA data among 3399 individuals.^25^ Using other imaging techniques such as ultrasound, quantification of subcutaneous abdominal tissue layers (SAT) into deep and superficial SAT and their functional differences have been reported.^26^ Studies involving postmenopausal women showed that abdominal obesity was characterized by increased CVD risk factors such as VLDL1‐TG and apoB production, hepatic fat and non‐HDL cholesterol, which has important implications for CVD risk in this group.^27^

##### Recruit-by-phenotype (RbP) studies

With the rich abundance of data within the baseline OBB characterization, participants can be selected based on pre-defined phenotypic traits (Table 1) for investigations of complex intermediary phenotypes. These include both *in vivo* physiological studies and case-control studies. Several *in vivo* studies using OBB have aimed at understanding adipose tissue biology, investigations into the T2D- and CVD-protective properties of gluteofemoral fat, and fatty acid trafficking. Participants have been selected to take part in complex protocols to study the metabolic physiology of the femoral adipose tissue depot.^28^ Using stable isotope-labelled metabolic tracers combined with arterio-venous sampling techniques, it has been found that: (i) muscle and adipose tissue handle fatty acid uptake very differently;^29^ and (ii) gluteofemoral adipose depots exhibit lower lipolytic activity^30^ and, in relative terms, greater extraction of lipids from ectopic fat deposition. This could explain some of the CVD- and T2D-protective effects seen with expansion of this fat depot.^31–34^

Deep physiological characterization of patients with rare genetic conditions requires access to carefully matched healthy controls for which OBB participants have been used. Examples of this includes familial combined hyperlipidaemia (FCHL),^35^ Chuvash polycythaemia,^36^ PTEN mutations^37^ and extreme high bone mass.^38^ Equally, in common disorders where pair-matching is essential for study design, OBB participants have been recruited as controls for studies of polycystic ovary syndrome^39^^,^^40^ and insulin resistance.^41^

##### Recruit-by-genotype studies (RbG)

The first use of OBB for RbG studies was the *in vivo* physiological characterization of adipose tissue function according to *PPARG* Pro12Ala carrier status among 42 age- and BMI-matched individuals. The matching for BMI was done to isolate the effect of metabolic phenotype by the PPARG genotype from a potential adiposity effect. Obese individuals carrying the T2D-protective Ala12 variant have higher adipose tissue blood flow than Pro12 carriers.^42^ The apolipoprotein-E (*APOE)* epsilon 4v variant is a risk gene variant for Alzheimer’s disease, which has been investigated for brain blood flow in relation to memory testing in age- and sex-matched participants from OBB.^43^ The physiological consequences of a *PPP1R3A* gene variant, identified in relation to digenic inheritance of partial lipodystrophy,^44^ was tested using the RbG concept.^45^ Besides metabolic disorders, the availability of large genotype data has also enabled the use of OBB in the investigation of other diseases. Using the RbG approach, we recently showed a protective homozygous trait for autoimmune diseases among carriers of tyrosine kinase-2 (TYK2).^46^

An updated list of publications from OBB is available at [https://scholar.google.co.uk/citations?hl=en&user=xPs_QwMAAAAJ].

## What are the main strengths and weaknesses? 

The strength of the cohort is in the triumvirate of detailed baseline characterization of a large random healthy population, the density of the genomic characterization and the recall capability. The cohort is not designed as a prospective follow-up cohort, and the phenotypic baseline characterization is dominated by metabolic measurements. The age range is limited to 30–50 years, and people with overt disease are excluded. We acknowledge that exclusion of T2DM and CVD cases enriched for genotypes of interest may introduce spurious associations due to collider effect and selection bias, particularly in genetic association studies and GWAS.^47^^,^^48^ Care would be taken to use appropriate statistical methods to account for such bias. However, these effects are likely to be reasonably small with the upper age limit being 50 years in the cohort.

## Can I get hold of the data? Where can I find out more?

The OBB is open for collaborative studies with academic and commercial partners after research protocols have been accepted by the OBB steering committee. Rules of engagement and contact with the OBB team can be found on the website [www.oxfordbiobank.org.uk].

## Funding

This work was supported by the British Heart Foundation from 2000 to 2003, by the Wellcome Trust from 2003 to 2009 and by the NIHR Oxford Biomedical Research Centre and the National NIHR Bioresource from 2007.
